# Testing the relationship between mimicry, trust and rapport in virtual reality conversations

**DOI:** 10.1038/srep35295

**Published:** 2016-10-14

**Authors:** Joanna Hale, Antonia F. De C. Hamilton

**Affiliations:** 1 University College London, UK

## Abstract

People mimic each other’s actions and postures during everyday interactions. It is widely believed this mimicry acts as a social glue, leading to increased rapport. We present two studies using virtual reality to rigorously test this hypothesis. In Study 1, 50 participants interacted with two avatars who either mimicked their head and torso movements at a 1 or 3 second time delay or did not mimic, and rated feelings of rapport and trust toward the avatars. Rapport was higher towards mimicking avatars, with no effect of timing. In Study 2, we aimed to replicate this effect in a pre-registered design and test whether it is modulated by cultural ingroup-outgroup boundaries. Forty participants from European or East Asian backgrounds interacted with four avatars, two of European appearance and two of East Asian appearance. Two avatars mimicked while the other two did not. We found no effects of mimicry on rapport or trust ratings or implicit trust behaviour in a novel maze task, and no effects of group status or interactions. These null results were calculated in line with our pre-registration. We conclude that being mimicked does not always increase rapport or trust, and make suggestions for future directions.

Humans mimic each other in many ways. We unconsciously imitate the postures^1^,^2^, facial expressions^3^,^4^, gestures^2^,^5^, moods and emotions^6^,^7^ and speech^7^,^8^ of other people. One major area of mimicry research focuses on the unconscious imitation of postures, gestures and mannerisms, termed behavioural mimicry^9^,^10^. Mimicry therefore differs from deliberate, goal-directed imitation (for other meanings see ref. 11). Mimicry and imitation emerge during infancy and have important roles for social learning and affiliation^12^,^13^. It is widely believed that mimicking another person has positive consequences for the social interaction^9^,^10^,^14^,^15^. Therefore mimicry has been described as a ‘social glue’^16^,^17^ or ‘honest signal’^18^ and has been advocated as a strategy for business and personal interactions, as well as teaching and therapy^1^,^19^,^20^. Understanding how people unconsciously detect when they are being mimicked also has important implications for our theories of social cognition. However, it is not yet clear if a person being mimicked responds to general contingency^21^ and predictability^22^,^23^ in the mimicker’s behaviour, or if the brain contains a specific mimicry detection mechanism (reviewed in ref. 11).

For all these reasons, it is important to probe how people respond to being mimicked in detail and with precise experimental control. Many previous studies of this topic have used naturalistic situations in which a confederate is trained to subtly mirror the movements and posture of a naïve participant during a social task such as describing photos to each other^2^,^14^. Headline results from these paradigms suggest that mimicry increases prosocial behaviour^14^,^24^,^25^ and may lead to greater liking^26^,^27^ and trust towards the mimicker^28^, though these results may be modulated by other factors^27^,^29^,^30^. However, there are several reasons to be cautious of accepting these naturalistic studies of mimicry at face value. First, both effect sizes and experimental power in many previous studies have been small, and false-positives may be present in the literature^11^. Second, many behaviours may naturally vary alongside mimicry, such as eye contact, smiles and the contingency between two people. It is not always clear if a confederate can accurately change her mimicry behaviour without also changing these other behaviours. Third, even well trained confederates lack control over the exact timing and matching precision of their movements. Finally, confederate behaviour may be implicitly affected by knowledge of the experimental condition or the cognitive demand of the task instructions^31^.

A strong test of the claim that mimicry itself leads to positive social consequences can come from virtual reality (VR), where every parameter of a social interaction can be precisely controlled. People react towards avatars similarly to real people^32^,^33^, so virtual reality is becoming an increasingly popular tool in social psychology and neuroscience. Bailenson and Yee^34^ generated mimicry in virtual reality, by tracking participant’s head movement and applying the same movement after a delay to an avatar. In the control condition, movements from a previous participant are applied to the avatar instead. This VR approach allows researchers to ‘reverse-engineer’ social interactions and determine precisely which parameters are essential for positive social effects^35^. It can thus provide a much stronger test of the ‘social glue’ hypothesis of mimicry than a confederate study.

Virtual mimicry is still a relatively new approach and has yielded mixed results. Bailenson and Yee^34^ found that virtual mimicry led to positive impressions of the avatar, consistent with earlier findings. However, other studies have not replicated their findings consistently^36^,^37^. If being mimicked by avatars does lead to increased rapport and trust, this provides a strong test of the social glue hypothesis and an important tool for future studies of the cognitive mechanisms which detect when someone is being mimicked.

The aim of the present project was to establish a paradigm in which participants can be mimicked by an avatar, and to use that to test the parameters which matter for this social interaction. We consider two types of parameters: *mimicry variables*, e.g. the timing and spatial form of mimicry, and *context variables*, e.g. social characteristics of the mimicker. In the first study we build on Bailenson and Yee’s method to implement virtual mimicry during an interactive photo description task. This matches the task commonly used in studies with confederate mimickers^2^,^14^. We test if and how changing the time delay between the participant’s action and the mimicry of the avatar alters the positive effects of being mimicked, to probe the mechanism of mimicry detection itself. Based on positive results from the first study, we designed and pre-registered a second study which uses the same paradigm to rigorously test whether the ingroup or outgroup membership of the mimicker modulates the effects of mimicry.

## Study 1

There is currently very little data about the cognitive mechanisms which spontaneously detect mimicry and trigger social responses towards a mimicker (for a review of possible cognitive mechanisms, see ref. 11). However, assuming there is a cognitive mechanism which responds when another person is mimicking me, we would expect this mechanism to have some temporal tuning window, whereby mimicry at short delays is easier to detect or has stronger consequences than mimicry at longer delays. Such a window might also be tuned to the natural timing of mimicry as it occurs in social interactions. Despite the importance of timing, precise data on this factor are limited^9^. Some claim that mimicry naturally occurs within a 2–5 s time window^15^,^30^, and this timescale has been used when training confederates^30^. However, others have adopted wider timescales ranging from zero^2^ to ten seconds^31^, and many studies do not report a timescale for mimicry. Therefore, it is unclear how the timing may impact the response to being mimicked. Preliminary evidence suggests people find it easier to deliberately detect virtual mimicry at a one-second time delay, compared to two, four or eight seconds^38^. However, no studies have formally tested whether the timing of mimicry affects positive consequences of being mimicked, such as feelings of rapport and trust. Tentatively, we would predict that if mimicry is easier to detect at shorter time delays, then a closer temporal coupling between mimicker and mimickee might increase the saliency and impact of mimicry.

**Figure 1:**
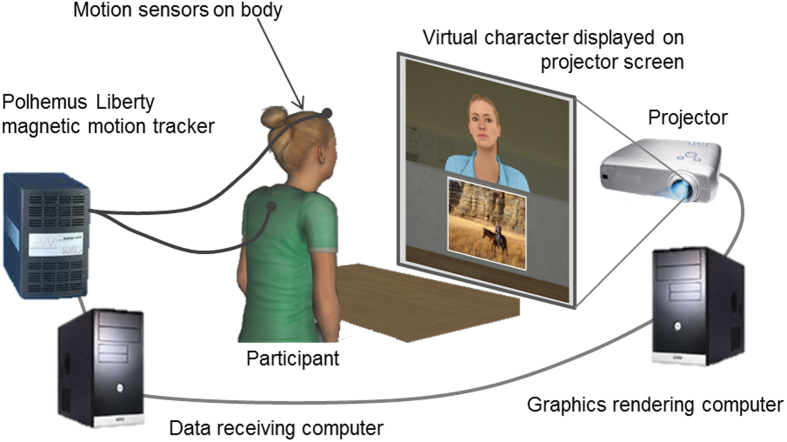
Virtual mimicry system. The participant was fitted with two motion tracking sensors which recorded their head and torso movements. The movement was mirrored by an avatar after a specified time delay. The avatar was displayed on a projector screen. This image was created using Vizard virtual reality software (WorldViz Inc, Version 5.4, http://www.worldviz.com/virtual-reality-software-downloads/).

Virtual mimicry is an ideal method for exploring this question, since avatars can be programmed to repeat the participant’s actions after a precise, constant time delay. Building on Bailenson and Yee’s^34^ approach, we implemented a virtual reality version of an interactive photo description task commonly used in confederate studies^2^,^15^. In this task, the participant and an avatar take turns to describe photographs to each other, giving the feeling of interaction without a full conversation (Fig. 1). We used two avatars: one mirrors the head and torso movements of the participants after a specific delay (1 second or 3 seconds); the other avatar shows pre-recorded natural head and torso movements without mimicry. Participants interacted with the mimicking and non-mimicking avatars one after the other, in a within subjects design. The time-delay of mimicry was a between-subjects factor. After interacting with each avatar, participants completed a number of ratings to evaluate their feelings about that avatar, including rapport, trust, similarity, the smoothness of the interaction and self-other overlap^39^, i.e. feelings of closeness towards others. A co-presence questionnaire was used to evaluate the realism of the VR^40^,^41^. At the end of the study, participants were carefully debriefed to determine if they consciously detected mimicry.

In this initial, exploratory study we aimed to test if there were any positive consequences of mimicry, to replicate previous results. We also tested if mimicry with a 1 second delay has stronger (or weaker) social effects than mimicry with a 3 second delay. Finally, we tested if conscious detection of mimicry varies between the 1 second and 3 second delay groups. Based on previous virtual mimicry research, we expected more participants would consciously detect mimicry at the shorter time delay. We also predicted that mimicry would have more positive effects at 1 s delay versus 3 s delay.

## Results

### Excluded data

Data were excluded from one participant in the 1 s group due to technical failure of the motion tracker.

### Mimicry detection

In the 3 s mimicry group, one participant out of 26 (3.8%) detected the mimicry manipulation. In the 1 s group, 11 out of 36 (30.5%) detected mimicry. The detection rate was significantly greater in the 1 s group than the 3 s group (*χ*^2^(1) = 6.9, *p* < 0.01). All detectors were excluded from the analyses. Thus, the remaining sample was *N* = 25 in each group.

### Co-presence

We averaged the four items from the co-presence questionnaire into one co-presence score (Cronbach’s alpha = 0.72). The median social presence score was 4.45 (*M* = 4.43, *SD* = 1.15, *Range* = [1.75, 6.75]) on a scale from 1 to 7, which is very similar to levels found in more immersive virtual reality experiments using a comparable rating scale^40^,^41^. Including co-presence as a covariate did not substantially change any of the results reported in the main analyses below (see Table S2, supplementary information, section 5).

### Ratings

Participants rated their feelings of rapport, trust and similarity towards each avatar, as well as the smoothness of the interaction. They also rated feelings of self-other overlap towards the specific avatar, avatars in general, their best friend and other people in general.

For each dependent variable, we conducted a two-way mixed-design ANOVA to test the within-participants effect of mimicry condition (mimicry vs. non-mimicry) and the between-participants effect of time delay (3 s vs. 1 s). The results are reported in Table 1. We found a significant main effect of mimicry on rapport in the expected direction (*p* = 0.02), but the Bayes factor for this effect was BF_01_ = 0.34, which is only weak evidence^42^. There were no other significant effects of mimicry or time delay, and no significant interactions, with most Bayes factors for these tests in favour for the null hypothesis (see Table S3, supplementary information, section 5).

To further explore the relationship between rapport and mimicry time delay, we calculated the effects of mimicry on rapport for the 1 s and the 3 s groups separately. We found that there was a significant effect of rapport in the 3 s condition (*t*(24) = 2.15, *p* = 0.04, *d* = 0.49) but not in the 1 s condition (*t*(24) = 1.39, *p* = 0.18, *d* = 0.36). There was only weak evidence for the effect in the 3 s group (BF_01_ = 0.89) and the effect in the 1 s group favoured the null hypothesis (BF_01_ = 2.01).

## Discussion

In this study, we developed a new virtual mimicry paradigm and investigated whether the timing of mimicry affects how people respond to being mimicked. We programmed avatars to mimic participants’ head and torso movements at a delay of three seconds or one second, compared to a non-mimicry condition. We measured social responses to being mimicked and how many participants consciously noticed being mimicked. These data allow us to consider if being mimicked by an avatar can act as social glue, and if the timing of the mimicry matters.

Our data give a reasonably positive response to the question of whether being mimicked leads to a more positive social evaluation. Participants rated a level of co-presence (feeling the avatars were really present with them) comparable to virtual reality experiments using more immersive technology^40^,^41^, suggesting our system achieved a strong degree of realism. Participants also gave higher rapport ratings to avatars who mimicked compared to those who did not. This is consistent with previous research using virtual and human mimickers^2^,^27^,^34^ and supports the idea that mimicry can act as a social glue even in tightly controlled VR settings. However, there are some caveats to this finding. First, we did not find effects of mimicry on any of our other dependent measures (trust, similarity, interaction smoothness and self-other overlap). It could be that the outcomes we chose to measure are more fragile than we expected; other studies have also failed to find statistically significant effects of imitation on trust^35^,^36^, similarity^43^ and self-other overlap^44^. Second, the effect on rapport which we did find was small (η_p_^2^ = 0.10) and would not meet a Bonferroni correction. Bayesian analysis indicates our data provide weak or anecdotal evidence for the effect of mimicry on rapport. With this in mind, we developed Study 2 as a pre-registered experiment to test the positive effects of mimicry more robustly.

The data gives conflicting accounts of the role of timing in mimicry. We found a significantly higher rate of mimicry detection at a delay of 1 s compared to 3 s, consistent with^38^. This implies that the ability to spontaneously detect when someone else mimics me is tuned to particular timings. However, the present study cannot distinguish if this process is sensitive to any contingent behaviour^21^ or specifically mimicry. After people who consciously detected mimicry were excluded from the analysis, we did not find any significant differences in social ratings between those who experienced mimicry with a 3 second delay compared to those experiencing a 1 second delay (although the effect of mimicry on rapport only reached significance in the 3 s group). This was surprising, and implies that there is a cognitive mechanism which responds positively to being mimicked outside awareness and is not tuned to a narrow time window. A more detailed review of possible cognitive mechanisms which respond to being mimicked is in ref. 11. Thus, the conscious detection of mimicry may be independent of how mimicry is used to evaluate another person. Though counterintuitive, this suggestion is consistent with results from a previous study which found the spatial correspondence between mimicker and mimickee movements affected mimicry detection but not the social evaluation of the mimicker^35^. Therefore, it is possible that the mechanism which triggers a positive response to being mimicked has a very broadly tuned time window for detecting such mimicry. This possibility would fit with evidence that people expect certain levels of contingency when interacting with another person^45^,^46^, as well as the observation that ‘positive’ mimicry effects may actually be driven by negative reactions to an absence of mimicry^15^. Future studies would need to test a wider range of time delays to explore this possibility.

## Study 2

In Study 1, we established that our VR mimicry paradigm can generate increases in rapport, similar to previous studies. The second study aimed to replicate our initial findings with a rigorous, pre-registered procedure and to further test if the social consequences of mimicry may be altered by group membership. To ensure validity, we tested a sample of 40 participants which gives enough statistical power to detect effects of a similar or smaller size to Study 1, and we pre-registered the experimental design, exclusion criteria and analyses^47^.

While it has been suggested that mimicry is an ‘honest signal’ used in all human societies^18^, it is not clear whether mimicry leads to increases in rapport and trust between people from different cultural groups^9^. People typically produce less mimicry towards others who they initially dislike^48^, outgroup members^49^,^50^ and others from a different race^51^. Some studies suggest being mimicked by an outgroup member depletes cognitive resources^29^ and leads to feeling cold^30^. On the other hand, a recent experiment found that being mimicked by an outgroup member (a Palestinian avatar) led participants (Jewish Israelis) to increase empathy towards their outgroup^52^. These mixed findings suggest outgroup mimicry can challenge people’s expectations but may also have positive social outcomes.

The present study tested whether mimicry leads to rapport and trust across individuals from the same social group (in-group pair) and different groups (out-group pair). To create in-group and out-group pairs, we used avatars with different apparent ethnicities and recruited participants from East Asia and Europe. Each participant met with 4 avatars who mimicked or did not and who did or did not appear to share the participant’s regional background in a 2 × 2 factorial within subjects design. Mimicry was implemented as in Study 1, with a 3 second time delay, and we continued testing until we had complete datasets from 40 participants without any detection of mimicry. This sample size was chosen based on a power analysis of study 1, in which we found a small effect of mimicry (η_p_^2^ = 0.10). We calculated that a sample size of 40 could detect the same size effect with power of 0.99, or a smaller effect size (η_p_^2^ = 0.06) with power of 0.95.

As in Study 1, participants completed the photo description task with each avatar, during which they were exposed to mimicry or non-mimicry behaviour. They rated likeability, trust and rapport towards each avatar, and we also measured behavioural trust using a new virtual maze task (see supplementary information). We predicted that participants should show a main effect of mimicry, with greater liking, trust and rapport towards avatars which mimicked. Furthermore, we predicted that this mimicry effect should be modulated by cultural group, such that all effects would be stronger for avatars from an in-group compared to those from an out-group. All the results we report here are as specified in our pre-registration^47^.

## Results and Discussion

### Excluded data

Six participants were excluded from analyses due to technical failure of the motion tracker. Eight out of the remaining 48 participants (16.6%) detected the virtual mimicry manipulation. All of the detectors were excluded from the analyses, so the remaining sample was *N* = 40. Four participants did not complete the virtual maze task with every avatar due to feelings of motion sickness, so their data was not included in analyses on that task.

### Co-presence

Participants completed the same co-presence ratings as in Study 1, once after interacting with ingroup avatars (*M* = 4.58, *SD* = 1.21) and once after interacting with outgroup avatars (*M* = 4.63, *SD* = 1.29). There was no significant difference in feelings of co-presence across ingroup and outgroup members (*t*(39) = 0.43, *p* = 0.67, *d* = 0.09). Therefore we averaged the two scores together. The median co-presence score was 4.75 (*M* = 4.61, *SD* = 1.19, *Range* = [1.63, 6.75]), consistent with Study 1 and other VR research^40^,^41^.

### Liking, rapport and trust ratings

We conducted two-way, repeated-measures ANOVAs to test the effects of mimicry and group membership on ratings of liking, rapport and trust towards each avatar. We did not find any significant main effects of mimicry or group membership, or any significant interaction effects (Table 2). There was a marginal effect of mimicry on trust in the expected direction: participants rated slightly more trust towards mimickers than non-mimickers.

### Virtual maze task

We conducted two-way, repeated-measures ANOVAs to test the effects of mimicry and group membership on how often participants approached an avatar in the virtual maze, and how often they followed an avatar’s advice (each expressed as a percentage of trials). We did not find any significant main effects of mimicry or group membership, or any significant interaction effects (Table 2). There was a marginal interaction between mimicry and group membership on how often each avatar was approached in the maze: participants approached the mimicking ingroup member on a greater percentage of trials than the non-mimicking ingroup member, whereas this trend was reversed for outgroup members.

### Exploratory analyses

In addition to the pre-registered analyses described above, we conducted further exploratory analysis of this dataset. Firstly, we carried out Bayesian ANOVAs testing the main effects of mimicry, group membership and the interaction between mimicry and group membership on each of our dependent variables (Table S4, supplementary information, section 6). The results showed that our data favour a null main effect of mimicry on ratings of liking, rapport and trust, as well as trust behaviour in the virtual maze (all BF_01_ > 1.6). The marginal interaction between mimicry and group membership on how often participant’s approached the avatars in the maze (reported above) had a Bayes factor of 5.37 in favour of the null hypothesis. Bayes factors favoured the null hypothesis for all main effects of group membership (all BF_01_ > 3.5) and all interactions between mimicry and group membership (all BF_01_ > 30).

We also carried out two further exploratory analyses to explore the role of co-presence and to test the effects of mimicry in data from the first two trials of the experiment, in case of fatigue effects occurring in the second half (see supplementary information, section 6). These exploratory analyses did not reveal any significant effects of mimicry or group membership, or any significant interactions.

### Summary

In this study we used VR to give participants an opportunity to be mimicked (or not) by an avatar from their ingroup or an outgroup. We measured the effect of mimicry on ratings of liking, rapport and trust, as well as implicit trust behaviour in a virtual maze task, and pre-registered all our analyses. With this rigorous test of the effects of virtual reality mimicry, we found null results. We did not find any significant main effects of mimicry or group membership, or any significant interactions, although there were some trends in the predicted directions. None of these trends would approach significance with a Bonferroni correction for five comparisons in place. In addition, Bayesian analysis showed that our data provides evidence in favour of the null hypothesis for all main effects of mimicry and group membership, and all interactions.

## General Discussion

The present paper reports two tightly controlled studies which used virtual reality to create a situation in which participants’ head and torso movements were mimicked (or not) by an avatar during a semi-natural conversation. We used a variety of measures to determine how being imitated changed participants’ evaluation of and willingness to engage with the avatar. Study 1 found a weak positive effect of mimicry, whereby participants reported stronger rapport towards an avatar who mimicked them with either a 1 second or 3 second delay, compared to an avatar who did not mimic. Study 2 did not replicate this effect, and our pre-registered analyses found that being mimicked did not change trust, liking or rapport either within or across social groups. Given these two contrasting results, we first consider which results are more reliable, and then explore the implications for future studies of the consequences of being mimicked.

First we consider virtual mimicry leads to rapport and trust. In Study 1 we presented a new virtual mimicry paradigm and used it to explore the effect of manipulating the time delay in mimicry. Participants were significantly more likely to consciously notice that they were being mimicked when the time delay in mimicry was 1 second, compared to 3 seconds. Participants showed greater rapport towards a mimicking avatar than a non-mimicking avatar. However, there was only weak evidence for this effect, and we did not find any other significant effects of mimicry or time delay on ratings of trust, similarity, interaction smoothness or self-other overlap, or any significant interactions between mimicry and time delay. Contrary to our prediction, the greater detection of mimicry at shorter time delays does not seem to correspond to a greater positive response towards the mimicker. Inspection of the data divided by time delay showed that mimicry increased rapport at 3 seconds delay but not at 1 second. This is noteworthy because it means that Study 2 did not use the wrong time delay to find positive effects of mimicry.

In Study 2, we used the same virtual mimicry method to test whether the effects of mimicry on rapport and trust depend on the group membership of the mimicker. Participants interacted with avatars who appeared to come from their home region (ingroup) or the other region (outgroup). We did not find any significant effects of mimicry or group membership on liking, rapport or trust towards the avatars. Of the two studies we report here, we consider Study 2 to be the more definitive. This is because Study 1 was an exploratory study with many data analysis options available, though we did not exploit all possibilities. In contrast, Study 2 was pre-registered with a single data analysis pathway, and provides a clear test of the prediction that mimicry leads to rapport and trust. This leads us to the conclusion that our results in Study 1 may have been false positives, and over both studies we cannot make the claim that being mimicked leads to changes in social evaluation.

Our data is in line with previous studies of mimicry using VR, which have reported decidedly mixed results. Bailenson and Yee’s^34^ virtual mimicry system had a positive effect on participants’ impression of an avatar, measured using a scale that taps into congeniality, knowledgeability and sincerity. Vrijsen *et al*.^5^ used the same paradigm in a study comparing socially anxious and non- anxious women’s responses. They did not report the main effect of mimicry, but report a near-significant positive effect of mimicry on how non-anxious women evaluated the avatar in terms of likeability and friendliness. Verberne *et al*.^36^ used the same mimicry algorithm and found inconsistent results across a range of measures. With one avatar, mimicry had a significant positive effect on liking and trust, but with another avatar the same mimicry manipulation did not lead to significant positive effects. Across both avatars there were no significant effects of mimicry on self-other overlap. In a subsequent study combining mimicry with similarity in goals and appearance, Verberne *et al*.^37^ also found inconsistent effects across different measures of trust. It is worth noting that ratings of liking, trust and self-other overlap also show inconsistent effects of mimicry in traditional research settings where human confederates were trained to mimic participants^11^. Therefore the positive social effects of being mimicked may be more subtle or fragile than is generally assumed. Overall, we conclude that the mimicry of participant head and torso movements alone by a virtual person does not lead to substantial increases in rapport and trust.

We turn now to the implications of the present research. The ‘social glue’ theory of mimicry^16^,^17^ suggests that non-conscious mimicry occurs across a wide range of behaviours including speech patterns, facial expressions, emotions, postures, gestures and mannerisms, and that mimicry of any of these behaviours can lead to positive social consequences such as rapport and interpersonal closeness. Virtual reality provides a strong test of this hypothesis, because it allows us to specifically control one type of mimicry while keeping all other social behaviours (speech, gaze, facial expression) exactly the same. Our findings thus cast doubt over a strong version of the social glue theory, in which all types of mimicry must have positive social effects. It seems that in the specific case of mimicking head and torso movements in a mirror fashion, neither a 1 second nor a 3 second delay leads to increases in liking, rapport or trust.

It could be argued that this result reflects a failure of VR rather than a failure of the social glue theory. We recognise that current VR is not perfect, and participants in the study are always aware that they are interacting with a virtual agent and not another real person. However, our co-presence scores of 4.45 (Study 1) and 4.75 (Study 2) on a scale from 1–7 are comparable to other VR studies. Such studies have successfully replicated psychology effects in the domain of body ownership^54^. VR interactions have been able to successfully generate joint attention^55^, proximity effects^56^ and audience effects^57^ among other phenomena. Thus, there is ample evidence that VR can generate socially realistic scenarios which replicate real-world psychological phenomena.

It is possible that our particular avatars lacked some critical behaviours, meaning that our VR scenario might not achieve a realistic replication of natural mimicry. Adding other social behaviours to our avatars, such as nodding and more facial expressions, might lead to stronger effects^58^. This might make the VR more similar to real-life social interactions, where a variety of other communicative signals may accompany mimicry of body posture, including emotional imitation^59^,^60^, turn-taking^18^,^60^ and eye-contact^61^. Traditional studies have attempted to control for the confounding effect of such cues by videotaping and rating mimickers’ behaviour^28^,^31^,^48^, but it is likely that some extra cues still accompany mimicry in naturalistic paradigms. Therefore by controlling all other social cues, virtual mimicry may not achieve as large effect sizes as naturalistic mimicry. It is also possible that mimicry may have different effects in the context of our structured conversation task, compared to other contexts involving different levels of interaction (e.g. a free conversation^2^,^14^) and different external cues (e.g. a negotiation agenda^28^). Finally, it is possible that a larger set of avatar stimuli might be needed find mimicry effects in a cross-cultural context, as these effects could be obscured by task effects associated with using a small number of avatars. However, in order to test the claim that mimicry itself leads to positive social effects, VR provides the strictest test without other interfering signals. Although there have been some positive results from VR scenarios which support the social glue claim, our data provides evidence in favour of a null effect of virtual mimicry on rapport (BF_01_ = 3.74, Study 2).

Though our results do not show that being mimicked leads to increases in rapport, they do suggest people can spontaneously detect being mimicked. In study 1, 30.5% of participants detected mimicry at 1 second and 3.8% at 3 seconds. In study 2, 16.6% of participants detected mimicry at 3 seconds. The mimicry implemented in these studies was a subtle mimicry of head and torso movement only, and we were careful not to suggest in any way that our study involved mimicry. Thus, we provide evidence that some cognitive mechanism is able to recall the pattern of head movements which a participant has made over at least 3 seconds and match that pattern to the behaviour of the avatar at a later point, leading to spontaneous detection. Due to the small number of people detecting mimicry and the possibility that detectors would show ‘demand characteristics’ in when rating the avatars, we were unable to analyse data from these participants in detail. However, it would be very interesting to test the mimicry detection mechanism in more detail in future, for example, to determine its tuning and whether it is sensitive to any type of contingency^21^ or only mimicry.

## Conclusions

Overall, we suggest our results should lead to caution in accepting the dominant view that being imitated by another person always leads to rapport. We cannot overturn a large body of work based on two studies, but rather we emphasise the fragile effects of being mimicked, and suggest that further work with larger sample sizes and rigorous methods will be needed to determine if mimicry really is a social glue. Further exploration and quantification of natural mimicry behaviour during social interactions would also be valuable in building better VR mimicry in the future.

## Methods

### Study 1

#### Participants

Sixty three participants (44 female, *M*_age_ = 26) were recruited through email advertisements. All participants gave written informed consent and received £7.50 payment for 1 hour. Recruitment continued until we obtained an equal number of participants in each group who did not consciously detect the mimicry manipulation. There were a total of 26 participants in the 3 s group and 37 participants in the 1 s group. Ethical approval was obtained from the UCL Institute of Cognitive Neuroscience Ethics Chair and the Ministry of Defence Research Ethics Committee. The methods were carried out in accordance with the approved guidelines. All participants gave written informed consent to take part.

#### Virtual Mimicry Algorithm

We prepared three avatars for the experiment. The first avatar was only used for practising the experimental tasks. The main two avatars in the experiment were females named Anna and Becky. The avatars were programmed to blink, make eye contact and make pre-recorded speech triggered by the computer program (for full details see supplementary information, section 1).

We recorded participants’ movements using a Polhemus magnetic motion tracking device (Polhemus Inc., Vermont). Participants were fitted with sensors on their forehead and upper back. The VR environment and all the avatars were animated in Vizard (WorldViz Inc).

In the mimicry condition, the participant’s head and torso movement was mirrored by an avatar presented on the projector screen (Fig. 1), after a specified delay. In addition, we imposed a restriction so the pitch (up/down tilt) of the participant’s head would only be mimicked within a range of 0 to −10 degrees from neutral. This was to avoid mimicry of upward head tilts, which appeared unnatural during piloting.

In the non-mimicry condition, the avatar was animated using pre-recorded movement from a pilot participant being mimicked. We used this pre-recording for all participants, instead of ‘yoking’ each participant to the previous one^34^,^53^, to ensure that an unusual behaviour in one participant would not interfere with the experience of the next participant.

#### Experiment procedure

On arriving in the lab, participants were told they were taking part in a virtual communication study and completed a consent form. They sat at a desk in front of a projector screen and Polhemus motion tracking sensors were fixed to their head and back. A short calibration was performed to allow the VR software to map the participant’s behaviour to an, and then the study began.

The participant practiced the task sequence detailed below. Then they completed the task sequence with Anna and then Becky. Either Anna or Becky mimicked the participant (mimicry condition), while the other avatar did not mimic (non-mimicry condition), in a counterbalanced fashion.

##### Mimicry induction: photo description task

The participant met each avatar in the virtual world and completed a photo description task with them (for full details, see supplementary information, section 1). Each avatar appeared as a life-sized person seated on the other side of the desk, facing the participant. The desk and walls of the lab appeared to extend into the virtual space. In the task, the avatar and participant would take 5 turns describing a photo to the other for 30 seconds. The avatar greeted the participant and smiled at the start, then began the task by describing a photo the participant could not see. At the end of their description, the avatar smiled again and then it was the participant’s turn to describe a photo, which was displayed on a virtual barrier between the participant and the avatar (Fig. 1). At the end of the task, the virtual barrier was raised up to occlude the avatar.

##### Ratings and questionnaires

Following the photo description task, the participant used a sliding scale based on the Inclusion of Other in the Self scale (IOS)^39^ to rate how much overlap they felt with their best friend (used as a reference point), the avatar from the photo task, s in general, and other people in general. Next, the participant rated the smoothness of the interaction and their feelings of rapport, trust and similarity on a continuous scale from ‘strongly disagree’ to ‘strongly agree’ (for all items see supplementary information, section 2).

At the end of the experiment, the participant rated the level of co-presence they experienced during the study on a 4-item scale from 1 to 7 (see supplementary information, section 2).

##### Debriefing

The participant provided written and verbal answers to a series of questions to determine whether they had noticed the mimicry manipulation or guessed the purpose of the experiment.

### Study 2

#### Participants

Participants completed a screening questionnaire to ensure they were suitable for the study (see supplementary information, section 3). Fifty-four participants (37 female) out of 236 total respondents were selected to take part in the study. We only selected participants if their answers to the screening questionnaire indicated they self-identified strongly with one of the groups chosen for the study. We included (1) individuals who identified strongly as European and (2) individuals who identified strongly as East Asian, had spent less than 1 year in the UK, and had spent most of the last 10 years in an East Asian country. The extra criteria for the East Asian group aimed to minimise overlap caused by recruiting and conducting the experiment in a European country. Ethical approval was obtained from the UCL Institute of Cognitive Neuroscience Ethics Chair and the Ministry of Defence Research Ethics Committee. The methods were carried out in accordance with the approved guidelines. All participants gave written informed consent to take part.

#### Virtual mimicry

We used the same equipment and algorithm as Study 1 to generate virtual mimicry.

**Figure 2:**
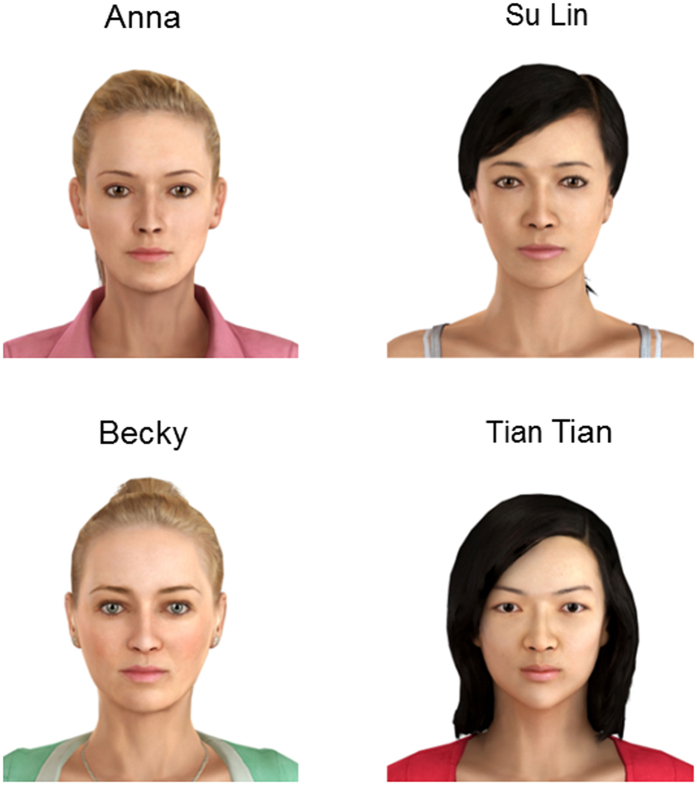
Avatar appearances. This image was created using Vizard virtual reality software (WorldViz Inc, Version 5.4, http://www.worldviz.com/virtual-reality-software-downloads/).

We prepared four avatars for the study (Fig. 2). We used the same Anna and Becky avatars as Study 1, because they had European appearances and their voices were recorded by volunteers with native British accents. The other two avatars, named named Su Lin and Tian Tian, have an East Asian appearance and their voices were recorded by volunteers speaking English with native Chinese accents. The avatars were programmed to blink, make eye contact with the participant and speak according to pre-recorded sound files, as in Study 1.

#### Virtual maze task

In this study we introduced a virtual maze task to measure behavioural trust (see supplementary information, section 4).

#### Experiment Procedure

Each participant sat at the desk in front of the projector screen, and the experimenter fitted the participant with motion tracking sensors. First the participant practiced the photo description task with the practice avatar from Study 1, who never mimicked them.

**Figure 3:**
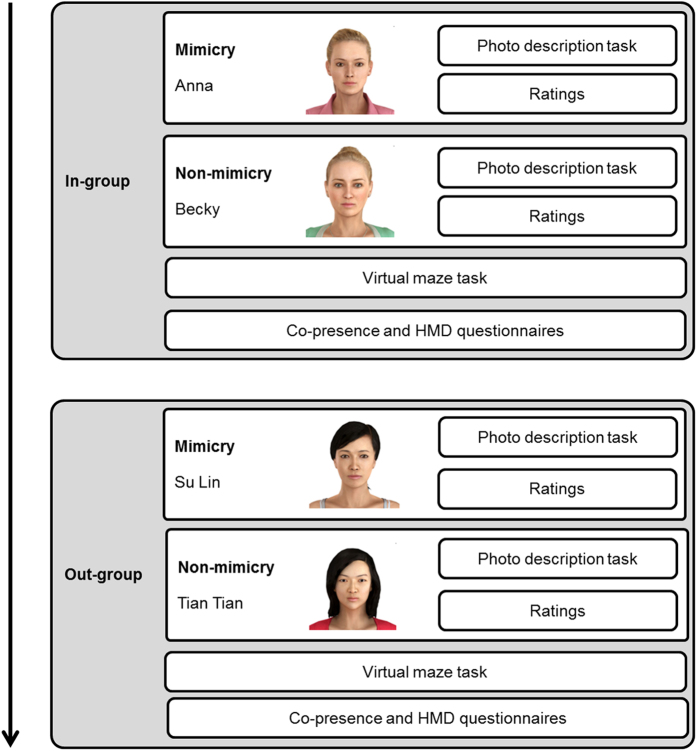
Experiment procedure, showing an example order of conditions. Group and mimicry were counterbalanced across participants. This image was created using Vizard virtual reality software (WorldViz Inc, Version 5.4, http://www.worldviz.com/virtual-reality-software-downloads/).

Following the practice, the participant completed the four experimental conditions in a counterbalanced order. The conditions were: (1) mimicry from an in-group member, (2) non-mimicry from an in-group member, (3) mimicry from an out-group member and (4) non-mimicry from an outgroup member. Half the participants met avatars from their in-group first, and the other half met avatars from their out-group first. For half the participants, the first avatar in the group mimicked them, and for the other half the second avatar in the group mimicked. The order of avatars in each group was always Anna then Becky, and Su Lin then Tian Tian. An example order of conditions is given in Fig. 3, which summarises the experiment procedure.

First, the participant completed the mimicry induction (photo description task) with the first avatar (e.g. Anna), as in Study 1. During the photo description task, the avatar either mimicked the participant’s movements after a 3 s delay, or performed pre-recorded movements.

Half-way through the photo description task, the participant was prompted to rate how much they agree that with the statement *‘I think Anna is very likeable’* on a continuous scale from strongly disagree to strongly agree. At the end of the photo description task, the participant rated their feelings of rapport and trust as in Study 1. Then the participant repeated the photo description task and ratings with the second avatar (e.g. Becky). The first two avatars were always a mimicker and non-mimicker from the same group.

After the participant completed the photo task and ratings with the first two avatars, the experimenter removed the motion sensors and prepared the participant for the virtual maze task. Then the participant completed the virtual maze task with the two avatars they had interacted with so far.

##### Questionnaires and debrief

Then the participant completed a questionnaire about their experience of co-presence, as in Study 1. Following the co-presence questionnaire, the participant repeated the entire procedure with the remaining two avatars (e.g. Su Lin and Tian Tian).

At the end of the experiment the participant completed the same screening questionnaire that we used at the recruitment stage. The participant also provided written and verbal answers to a series of questions to determine whether they had guessed the purpose of the experiment or noticed the mimicry manipulation.

## Additional Information

**How to cite this article**: Hale, J. and Hamilton, A. F. De C. Testing the relationship between mimicry, trust and rapport in virtual reality conversations. *Sci. Rep*. **6**, 35295; doi: 10.1038/srep35295 (2016).

## Supplementary Material

Supplementary Information

## Figures and Tables

**Table 1:** Effects of mimicry and time delay on ratings.

Measure	Mimicry *M* (*SD*)	Non-mimicry *M* (*SD*)	Main effect of mimicry			Main effect of time delay			Mimicry × time delay interaction		
			*F*(1, 48)	*p*	η_p_^2^	*F*(1, 48)	*p*	η_p_^2^	*F*(1, 48)	*p*	η_p_^2^
Rapport	0.69 (0.15)	0.63 (0.15)	5.54	0.02	0.10	0.63	0.43	0.01	0.002	0.97	>0.001
Trust	0.65 (0.14)	0.62 (0.15)	1.90	0.17	0.04	1.65	0.21	0.03	0.31	0.58	0.01
Similarity	0.55 (0.17)	0.51 (0.20)	1.52	0.22	0.03	1.22	0.28	0.03	0.04	0.85	0.001
Smoothness	0.67 (0.20)	0.66 (0.20)	0.006	0.94	>0.001	0.63	0.43	0.01	0.03	0.86	0.001
Overlap: specific avatar	0.42 (0.21)	0.41(0.19)	0.28	0.60	0.01	3.60	0.06	0.07	0.31	0.58	0.01
Overlap: avatars in general	0.39 (0.23)	0.40 (0.20)	0.50	0.50	0.01	2.50	0.12	0.05	0.12	0.73	0.002
Overlap: best friend	0.60 (0.26)	0.60 (0.26)	0.21	0.65	0.004	0.005	0.94	>0.001	0.09	0.77	0.002
Overlap: others in general	0.40 (23)	0.42 (0.23)	3.80	0.06	0.07	2.20	0.14	0.04	0.05	0.82	0.001

**Table 2:** Effects of mimicry and group membership on ratings and virtual maze task.

Measure	Ingroup		Outgroup		Main effect of mimicry			Main effect of group			Mimicry × group interaction		
	Mimicry *M* (*SD*)	Non-mimicry *M* (*SD*)	Mimicry *M* (*SD*)	Non-mimicry *M* (*SD*)	*F*(1, 48)	*p*	η_p_^2^	*F*(1, 48)	*p*	η_p_^2^	*F*(1, 48)	*p*	η_p_^2^
Liking rating	0.69 (0.21)	0.65 (0.21)	0.69 (0.18)	0.66 (0.17)	2.35	0.13	0.06	0.06	0.81	0.002	0.13	0.72	0.003
Rapport rating	0.72 (0.16)	0.70 (0.15)	0.70 (0.15)	0.69 (0.13)	1.06	0.31	0.03	1.35	0.25	0.03	0.25	0.62	0.006
Trust rating	0.68 (0.15)	0.66 (0.16)	0.70 (0.14)	0.65 (0.13)	3.81	0.06	0.09	0.04	0.84	0.001	0.43	0.52	0.01
Approach (maze)	0.86 (0.22)	0.80 (0.22)	0.80 (0.25)	0.84 (0.22)	0.19	0.67	0.005	0.05	0.82	0.001	3.08	0.09	0.08
Follow advice (maze)	0.64 (0.22)	0.61 (0.20)	0.62 (0.25)	0.64 (0.18)	0.04	0.85	0.001	0.06	0.81	0.002	0.37	0.55	0.01

## References

[b1] LaFrance M. & Broadbent M. Group Rapport: Posture Sharing as a Nonverbal Indicator. Group Organ. Manag. 1, 328–333 (1976).

[b2] Chartrand T. L. & Bargh J. A. The chameleon effect: The perception–behavior link and social interaction. J. Pers. Soc. Psychol. 76, 893–910 (1999). 10402679 10.1037//0022-3514.76.6.893

[b3] Bavelas J. B., Black A., Lemery C. R. & Mullett J. ‘I show how you feel’: Motor mimicry as a communicative act. J. Pers. Soc. Psychol. 50, 322–329 (1986).

[b4] Hess U. & Blairy S. Facial mimicry and emotional contagion to dynamic emotional facial expressions and their influence on decoding accuracy. Int. J. Psychophysiol. 40, 129–141 (2001). 11165351 10.1016/s0167-8760(00)00161-6

[b5] Dabbs J. M. Jr. Similarity of gestures and interpersonal influence. Proc. Annu. Conv. Am. Psychol. Assoc. 4, 337–338 (1969).

[b6] Hsee C. K., Hatfield E., Carlson J. G. & Chemtob C. The effect of power on susceptibility to emotional contagion. Cogn. Amp Emot. 4, 327–340 (1990).

[b7] Neumann R. & Strack F. ‘Mood contagion’: the automatic transfer of mood between persons. J. Pers. Soc. Psychol. 79, 211–223 (2000). 10948975 10.1037//0022-3514.79.2.211

[b8] Giles H. & Powesland P. F. Speech style and social evaluation. viii, (Academic Press, 1975).

[b9] Chartrand T. L. & Lakin J. L. The Antecedents and Consequences of Human Behavioral Mimicry. Annu. Rev. Psychol. 64, 285–308 (2013). 23020640 10.1146/annurev-psych-113011-143754

[b10] Lakin J. L. & Chartrand T. L. Using Nonconscious Behavioral Mimicry to Create Affiliation and Rapport. Psychol. Sci. 14, 334–339 (2003). 12807406 10.1111/1467-9280.14481

[b11] Hale J. & Hamilton A. F. de C. Cognitive mechanisms for responding to mimicry from others. Neurosci. Biobehav. Rev. 63, 106–123 (2016). 26877104 10.1016/j.neubiorev.2016.02.006

[b12] Uzgiris I. C. Two Functions of Imitation During Infancy. Int. J. Behav. Dev. 4, 1–12 (1981).

[b13] Over H. & Carpenter M. The Social Side of Imitation. Child Dev. Perspect. 7, 6–11 (2013).

[b14] van Baaren R. B., Holland R. W., Kawakami K. & van Knippenberg A. Mimicry and Prosocial Behavior. Psychol. Sci. 15, 71–74 (2004). 14717835 10.1111/j.0963-7214.2004.01501012.x

[b15] van Baaren R. B., Decety J., Dijksterhuis A., van der Leij A. & van Leeuwen M. L. In The social neuroscience of empathy (eds Decety J. & Ickes W.) 31–42 (MIT Press, 2009).

[b16] Lakin J. L., Jefferis V. E., Cheng C. M. & Chartrand T. L. The Chameleon Effect as Social Glue: Evidence for the Evolutionary Significance of Nonconscious Mimicry. J. Nonverbal Behav. 27, 145–162 (2003).

[b17] Dijksterhuis A. Why we are social animals: The high road to imitation as social glue. Perspect. Imitation Neurosci. Soc. Sci. 2, 207–220 (2005).

[b18] Pentland A. (Sandy). Honest Signals. (MIT Press, 2010).

[b19] Bernieri F. J. Coordinated movement and rapport in teacher-student interactions. J. Nonverbal Behav. 12, 120–138 (1988).

[b20] Scheflen A. E. The significance of posture in communication systems. Psychiatry J. Study Interpers. Process. 27, 316–331 (1964). 10.1080/00332747.1964.11023403 14216879

[b21] Heyes C. Automatic imitation. Psychol. Bull. 137, 463–483 (2011). 21280938 10.1037/a0022288

[b22] Kilner J. M., Friston K. J. & Frith C. D. Predictive coding: an account of the mirror neuron system. Cogn. Process. 8, 159–166 (2007). 17429704 10.1007/s10339-007-0170-2 PMC2649419

[b23] Friston K., Mattout J. & Kilner J. Action understanding and active inference. Biol. Cybern. 104, 137–160 (2011). 21327826 10.1007/s00422-011-0424-z PMC3491875

[b24] Ashton-James C. E., van Baaren R. B., Chartrand T. L., Decety J. & Karremans J. Mimicry and Me: The Impact of Mimicry on Self–Construal. Soc. Cogn. 25, 518–535 (2007).

[b25] Fischer-Lokou J., Martin A., Guéguen N. & Lamy L. Mimicry and propagation of prosocial behavior in a natural setting. Psychol. Rep. 108, 599–605 (2011). 21675573 10.2466/07.17.21.PR0.108.2.599-605

[b26] Kouzakova M., van Baaren R. & van Knippenberg A. Lack of behavioral imitation in human interactions enhances salivary cortisol levels. Horm. Behav. 57, 421–426 (2010). 20109459 10.1016/j.yhbeh.2010.01.011

[b27] Stel M., Rispens S., Leliveld M. & Lokhorst A. M. The consequences of mimicry for prosocials and proselfs: Effects of social value orientation on the mimicry-liking link. Eur. J. Soc. Psychol. 41, 269–274 (2011).

[b28] Maddux W. W., Mullen E. & Galinsky A. D. Chameleons bake bigger pies and take bigger pieces: Strategic behavioral mimicry facilitates negotiation outcomes. J. Exp. Soc. Psychol. 44, 461–468 (2008).

[b29] Dalton A. N., Chartrand T. L. & Finkel E. J. The schema-driven chameleon: how mimicry affects executive and self-regulatory resources. J. Pers. Soc. Psychol. 98, 605–617 (2010). 20307132 10.1037/a0017629

[b30] Leander N. P., Chartrand T. L. & Bargh J. A. You Give Me the Chills Embodied Reactions to Inappropriate Amounts of Behavioral Mimicry. Psychol. Sci. 23, 772–779 (2012). 22609538 10.1177/0956797611434535

[b31] Stel M., van Dijk E. & Olivier E. You Want to Know the Truth? Then Don’t Mimic! Psychol. Sci. 20, 693–699 (2009). 19422628 10.1111/j.1467-9280.2009.02350.x

[b32] Bailenson J. N., Blascovich J., Beall A. C. & Loomis J. M. Equilibrium Theory Revisited: Mutual Gaze and Personal Space in Virtual Environments. Presence Teleoperators Virtual Environ. 10, 583–598 (2001).

[b33] Garau M., Slater M., Pertaub D.-P. & Razzaque S. The Responses of People to Virtual Humans in an Immersive Virtual Environment. Presence Teleoperators Virtual Environ. 14, 104–116 (2005).

[b34] Bailenson J. N. & Yee N. Digital Chameleons Automatic Assimilation of Nonverbal Gestures in Immersive Virtual Environments. Psychol. Sci. 16, 814–819 (2005). 16181445 10.1111/j.1467-9280.2005.01619.x

[b35] Bailenson J. N., Yee N., Patel K. & Beall A. C. Detecting digital chameleons. Comput. Hum. Behav. 24, 66–87 (2008).

[b36] Verberne F. M. F., Ham J., Ponnada A. & Midden C. J. H. In Persuasive Technology (eds Berkovsky S. & Freyne J.) 234–245 (Springer Berlin Heidelberg, 2013).

[b37] Verberne F. M. F., Ham J. & Midden C. J. H. Trusting a Virtual Driver That Looks, Acts, and Thinks Like You. Hum. Factors J. Hum. Factors Ergon. Soc. 18720815580749, doi: 10.1177/0018720815580749 (2015). 25921302

[b38] Bailenson J. N., Beall A. C., Loomis J., Blascovich J. & Turk M. Transformed Social Interaction: Decoupling Representation from Behavior and Form in Collaborative Virtual Environments. Presence Teleoperators Virtual Environ. 13, 428–441 (2004).

[b39] Aron A., Aron E. N. & Smollan D. Inclusion of Other in the Self Scale and the structure of interpersonal closeness. J. Pers. Soc. Psychol. 63, 596–612 (1992).

[b40] Pan X., Gillies M., Barker C., Clark D. M. & Slater M. Socially Anxious and Confident Men Interact with a Forward Virtual Woman: An Experimental Study. PLoS ONE 7, e32931 (2012). 22509251 10.1371/journal.pone.0032931 PMC3324473

[b41] Friedman D. . A method for generating an illusion of backwards time travel using immersive virtual reality—an exploratory study. Percept. Sci. 5, 943 (2014). 10.3389/fpsyg.2014.00943 PMC4151165 25228889

[b42] Raftery A. E. Bayesian Model Selection in Social Research. Sociol. Methodol. 25, 111–163 (1995).

[b43] Reddish P., Fischer R. & Bulbulia J. Let’s Dance Together: Synchrony, Shared Intentionality and Cooperation. PLoS ONE 8 (2013). 10.1371/journal.pone.0071182 PMC3737148 23951106

[b44] Hogeveen J., Chartrand T. L. & Obhi S. S. Social Mimicry Enhances Mu-Suppression During Action Observation. Cereb. Cortex bhu016, doi: 10.1093/cercor/bhu016 2014). 24532320

[b45] Bigelow A. E. Infants’ sensitivity to familiar imperfect contigencies in social interaction. Infant Behav. Dev. 21, 149–162 (1998).

[b46] Bigelow A. E. Discovering self through other: Infants’ preference for social contingency. Bull. Menninger Clin. 65, 335–346 (2001). 11531130 10.1521/bumc.65.3.335.19852

[b47] Hale J. & Hamilton A. F. de C. Does group membership matter for how people respond to mimicry? (2015).

[b48] Stel M. . Mimicking disliked others: Effects of a priori liking on the mimicry-liking link. Eur. J. Soc. Psychol. 40, 867–880 (2010).

[b49] Bourgeois P. & Hess U. The impact of social context on mimicry. Biol. Psychol. 77, 343–352 (2008). 18164534 10.1016/j.biopsycho.2007.11.008

[b50] Yabar Y., Johnston L., Miles L. & Peace V. Implicit behavioral mimicry: Investigating the impact of group membership. J. Nonverbal Behav. 30, 97–113 (2006).

[b51] Johnston L. Behavioral Mimicry and Stigmatization. Soc. Cogn. 20, 18–35 (2002).

[b52] Hasler B. S., Hirschberger G., Shani-Sherman T. & Friedman D. A. Virtual Peacemakers: Mimicry Increases Empathy in Simulated Contact with Virtual Outgroup Members. Cyberpsychology Behav. Soc. Netw. doi: 10.1089/cyber.2014.0213 (2014). PMC4267545 25343577

[b53] Vrijsen J. N., Lange W.-G., Dotsch R., Wigboldus D. H. J. & Rinck M. How do socially anxious women evaluate mimicry? A virtual reality study. Cogn. Emot. 24, 840–847 (2010).

[b54] Maister L., Slater M., Sanchez-Vives M. V. & Tsakiris M. Changing bodies changes minds: owning another body affects social cognition. Trends Cogn. Sci. 19, 6–12 (2015). 25524273 10.1016/j.tics.2014.11.001

[b55] Schilbach L. . Minds Made for Sharing: Initiating Joint Attention Recruits Reward-related Neurocircuitry. J. Cogn. Neurosci. 22, 2702–2715 (2009). 10.1162/jocn.2009.21401 19929761

[b56] McCall C. & Singer T. Facing Off with Unfair Others: Introducing Proxemic Imaging as an Implicit Measure of Approach and Avoidance during Social Interaction. PLOS ONE 10, e0117532 (2015). 25675444 10.1371/journal.pone.0117532 PMC4326128

[b57] Slater M., Pertaub D. P. & Steed A. Public speaking in virtual reality: facing an audience of avatars. IEEE Comput. Graph. Appl. 19, 6–9 (1999).

[b58] Gratch J., Wang N., Gerten J., Fast E. & Duffy R. In Intelligent Virtual Agents (eds Pelachaud C. .) 125–138 (Springer Berlin Heidelberg, 2007).

[b59] Chartrand T. L., Maddux W. W. & Lakin J. L. In The *new* unco*ns*cious (eds. Hassin R. R., Uleman J. S. & Bargh J. A.) 334–361 (Oxford University Press, 2005).

[b60] Wallbott H. G. In Mutualities in dialogue (eds Markova I., Graumann C. F. & Foppa K.) 82–98 (Cambridge University Press, 1995).

[b61] Wang Y., Newport R. & Hamilton A. F. de C. Eye contact enhances mimicry of intransitive hand movements. Biol. Lett. 7, 7–10 (2011). 20427328 10.1098/rsbl.2010.0279 PMC3030861

