# Supplementary material for: Testing the relationship between mimicry, trust and rapport in virtual reality conversations
Source: Sci Rep. 2016 Oct 14;6:35295. doi: 10.1038/srep35295 (PMC5064448; doi:10.1038/srep35295)
Supplement: Supplementary Information [file srep35295-s1.pdf]

Supplementary Information for

Testing the relationship between mimicry, trust and rapport in virtual reality conversations

Joanna Hale and Antonia F. De C. Hamilton, University College London

1. Photo description task

The photo description task was used in Study 1 and Study 2 to induce mimicry. Apart from mimicry behaviour, we programmed each virtual character to describe photos to the participant and perform some other social behaviours to make the interaction seem more natural. Each virtual character was animated to smile and greet the participant at the start of the task. When it was the character's turn speaking, they moved their jaw according to the amplitude of a pre-recorded audio file of a volunteer describing a photo. The character alternated between tipping their head to look down at the photo they were describing (for 5-8s), and looking up at the participant with direct gaze (for 1-3s) throughout their turn. When it was the participant's turn speaking, the virtual character smiled once and then alternated between direct gaze (for 4-7s) and averted gaze (for 0.75-1.5s) throughout the participant's turn. The virtual character was programmed to blink every 2-5s.

We pre-recorded the descriptions made by each of the virtual characters. The descriptions for Anna and Becky (Study 1 and Study 2) were recorded for a previous virtual mimicry study in our lab. Two volunteers with native British accents came to the lab and their voices were recorded using headset microphones. The volunteers were asked to take turns describing a set of 10 photo stimuli to each other. They were told to speak for at least 30s per photo (timed by a researcher on a stopwatch) to ensure we obtained at least 30s of recorded description. We later edited each recording to exactly 30s in duration, so that each virtual character would speak for the full length of each turn in the experiment. First, the volunteers practiced describing the photos to each other. Then we recorded them while they described the same set of stimuli to each other (i.e. each volunteer described all 10 photos). During the experiment, the stimuli were randomly divided between Anna and Becky, so that each participant heard a random set of five descriptions from each character.

The descriptions for Su Lin and Tian Tian (Study 2) were recorded at a later date. Two volunteers with native Chinese accents came to the lab and we obtained recordings using the same procedure as above. Each volunteer described 10 stimuli; during the experiment these were randomly divided between Su Lin and Tian Tian.

2. Ratings and questionnaires

In Study 1, participants were asked to rate feelings of smoothness, rapport, trust and similarity after interacting with each virtual character. In Study 2, participants only rated rapport and trust. All ratings were made on a continuous scale from 'strongly disagree' (scored 0) to 'strongly agree' (scored 1). The items are given below (items marked with an asterisk were reverse scored). The order of the items within each category (e.g. rapport) was randomised for each participant.

Smoothness

I think the interaction with [Character] was smooth.
*I think the interaction with [Character] was awkward.

Rapport

I think [Character] is very likeable.
I think [Character] is very engaging.
I think [Character] is very kind.
*I think [Character] is very unfriendly.
*I think [Character] is very unpleasant.

Trust

I think [Character] is very trustworthy.
I think [Character] is very honest.
I think [Character] is very responsible.
*I think [Character] is very unreliable.
*I think [Character] is very insincere.

Similarity

I think [Character] is very similar to me.
*I think [Character] is very different from me.

Participants in Study 1 and Study 2 rated the level of co-presence they experienced during the study on a 4-item scale from 1 (not at all) to 7 (very much so). Items were presented in the following fixed order:

How much did you find yourself reacting to the avatars as real people, concerning your thoughts?

How much did you find yourself reacting to the avatars as real people, concerning your feelings and emotions?

How much did you find yourself reacting to the avatars as real people, concerning your physical responses (e.g. gestures, facial expressions)?

How much did you find yourself reacting to the avatars as real people, concerning your physiological responses (e.g. heart rate, sweat, blushing, etc.)?

3. Study 2 screening questionnaire

Participants in Study 2 completed the following screening questionnaire online so that we could select participants who were suitable for the study. Participants who were recruited for the study completed the same questionnaire at the end of the study to confirm their suitability had not changed. We included two groups of participants, with the following criteria:

European group

- The participant stated nationality was from a European country. (Q1)
- The participant rated the importance of nationality to their identity as at least 5 on a scale from 0 (extremely unimportant) to 6 (extremely important). (Q2)

East Asian group

- The participant stated nationality was from a European country. (Q1)
- The participant rated the importance of nationality to their identity as at least 5 on a scale from 0 (extremely unimportant) to 6 (extremely important). (Q2)
- The participant stated they had spent less than 1 year in the UK. (Q8)
- The participant stated they had spent most of the last 10 years in an East Asian country. (Q9)

Questionnaire

1. What is your nationality?
2. How much is your nationality important to your identity?

   Extremely unimportant 0 1 2 3 4 5 6 Extremely important

3. What is your ethnicity?
4. How much is your ethnicity important to your identity?

   Extremely unimportant 0 1 2 3 4 5 6 Extremely important

5. What is your first language(s)?
6. How much is your first language(s) important to your identity?

   Extremely unimportant 0 1 2 3 4 5 6 Extremely important

7. What other languages do you speak? Please list any languages you speak well enough to hold a conversation.
8. How many years have you lived in the UK? Please include time in the past as well as your current residence.
   - Less than 1 year
   - 1 - 5 years
   - 5 - 10 years
   - 10 years or more
9. Over the last 10 years, which country have you lived in for the most time?
10. Date of birth
11. Gender

4. Virtual maze task

In Study 2 we introduced a virtual maze task to measure behavioural trust. We have piloted and validated this task in other studies (Hale & Hamilton, *The virtual maze: A behavioural tool for measuring trust*, submitted). In the task, the participant navigates through a series of rooms (Fig. S1) and in each room they may ask for advice from virtual characters about which way to go next. The maze was displayed via an Oculus Rift DK2 head-mounted display (HMD). This device allows the participant to look around a virtual 3D space as if they are really there. The participant was provided with a joystick to move through the virtual space, similar to playing a video game. Participants first completed some practice to familiarise them with the 3D environment. Then they were instructed to find the way out of the maze through the least number of rooms possible. The computer program ensured that each participant continued until they had gone through twelve rooms (corresponding to twelve trials).

Virtual maze environment. The virtual maze was generated from a series of identical rooms (Figure 3) connected by short sections of corridor. The participant entered each room through a brown door; at the far end of the room, there was a red door and a blue door. In each room there were also two semi-transparent ‘hologram chambers’, where virtual characters (e.g. Anna and Becky or Su Lin and Tian Tian) appeared as ‘holograms’ from outside the maze. Whenever the participant got close to a hologram chamber, there was a sound effect and the chamber became more transparent. At the same time, the character inside the chamber would spin to face the participant and deliver some verbal advice. Participants were able to navigate the virtual environment by using a joystick.

Virtual character advice. The two virtual characters (e.g. Anna and Becky or Su Lin and Tian Tian) were programmed to advise the red door in half the trials and the blue door in the other half. They were also programmed to advise the same door as each other in half the trials and different doors in the other half. In order to generate the verbal advice stimuli, we pre-recorded twelve scripted phrases. Then we paired the phrases in order to create twelve combinations of advice stimuli (Table S1). The order of the stimulus combinations was randomised for each participant. Note that the participant would only receive an advice stimulus if they approach a character for advice. Therefore, some participants may not have received both parts of every stimulus combination.

Table S1. Virtual maze task advice stimuli

Trial	Combinations of verbal advice from virtual characters	
1	I think you should try the blue door.	I think you should go through the red door.
2	I think you go through the blue door this time.	I think it’s the red door this time.
3	It’s the blue door, I think.	It’s red this time, I think.
4	I think you should try the red door.	I think you should go through the blue door.
5	I think you go through the red door this time.	I think it’s the blue door this time.
6	It’s the red door, I think.	It’s blue this time, I think.
7	It’s blue this time, I think.	It’s the blue door, I think.
8	I think it’s the blue door this time.	I think you go through the blue door this time.
9	I think you should go through the blue door.	I think you should try the blue door.
10	It’s red this time, I think.	It’s the red door, I think.
11	I think it’s the red door this time.	I think you go through the red door this time.
12	I think you should go through the red door.	I think you should try the red door.

Trial procedure. Going through one room corresponded to completing one trial. Each participant completed twelve trials in total. In each trial, they had to make a choice about whether to proceed through the blue door or the red door. The participant was able to approach neither, one or both characters to receive advice about which door to choose, although this was not explicitly instructed. If approached, the characters randomly delivered uncertain advice about which door the participant should choose (e.g. *'It's blue this time, I think'*). There was no 'correct' door on each trial. Instead, the maze was completed after twelve trials in which the participant approached at least one character. If a participant asked neither character, that trial was recorded but did not count towards the requisite twelve trials. This ensured we had twelve trials in which the participant received some advice about which way to go, thus providing data about how much they trusted that advice.

Outcome measures. On each trial we recorded whether the participant approached each character for advice or not, and whether they followed the advice of each character or not. This provided two measures of trust: (1) how often each character was approached (expressed as a percentage of trials) and (2) how often each character's advice was followed (expressed as a percentage of trials).

VR Questionnaire. At the end of the maze task participants were asked to complete a short questionnaire about their experience in the virtual maze and whether they experienced any symptoms of motion sickness, headache or eye strain. Data from the VR questionnaire were not included in the present study.

The following items were answered on a 7-point scale from 'strongly disagree' to 'strongly agree':

During the maze task, the virtual space felt like the real world for me
During the maze task, I often thought I was just sitting in a laboratory
During the maze task, my behaviour was the same as if the situation were real
During the maze task, my thoughts were the same as if the situation were real

The participant was also asked to 'please tick any of the following that apply to you':

I feel motion sick
I feel queasy
I have a headache
My eyes are strained

Participants who reported any of the symptoms above were not required to continue with the study.

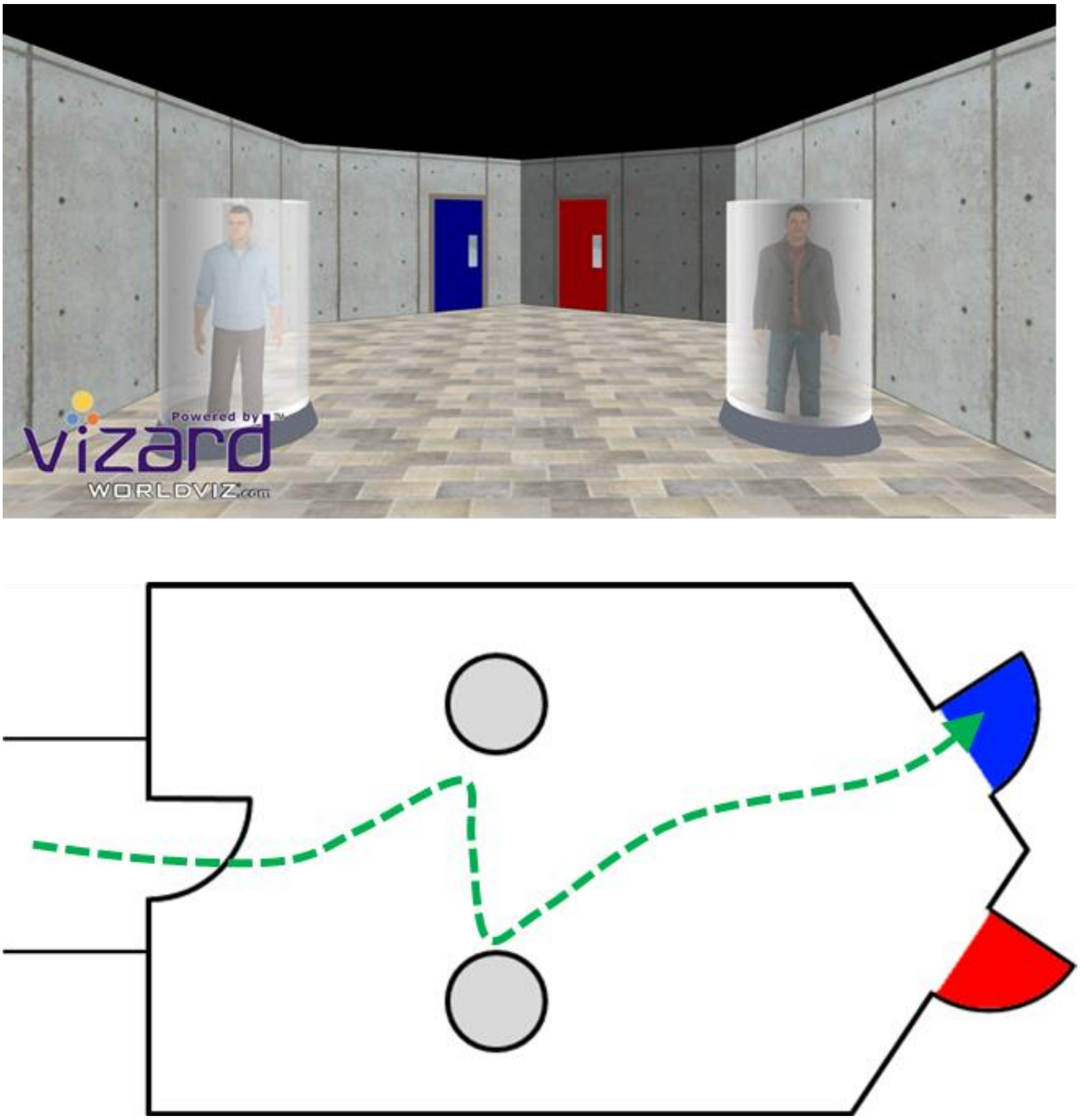

Figure S1. Screenshot and plan of each room in the virtual maze. A series of identical rooms made up the virtual maze. The grey circles on the plan indicate the position of the virtual characters. The dashed green line illustrates a possible route through the room. Going through one room corresponded to completing one trial. This image was created using Vizard virtual reality software (WorldViz Inc, Version 5.4, http://www.worldviz.com/virtual-reality-software-downloads/).

5. Study 1 supplementary analyses

In order to check whether participants' experience of co-presence modulated any of the effects in Study 1, we carried out a series of ANCOVAs, controlling for co-presence as a covariate. There were no significant main effects of mimicry on any of our dependent variables when co-presence was included as a covariate in the analysis (Table S2). There were also no significant main effects of time delay or any significant interactions between mimicry and time delay.

Table S2. Effects of mimicry and time delay on ratings, controlling for levels of co-presence.

	Main effect of mimicry			Main effect of time			Mimicry x time interaction		
Measure	*F*(1, 48)	*p*	η_p^2	*F*(1, 48)	*p*	η_p^2	*F*(1, 48)	*p*	η_p^2
Rapport	.48	.49	.01	.49	.49	.01	.001	.97	>.001
Trust	1.96	.17	.04	1.46	.23	.03	.46	.50	.01
Similarity	1.08	.30	.02	1.02	.32	.02	.08	.78	.002
Smoothness	1.13	.29	.02	.48	.49	.01	.06	.81	.001
Overlap: specific avatar	.07	.80	.001	3.48	.07	.07	.28	.60	.006
Overlap: avatars in general	.04	.84	.001	2.41	.13	.05	.13	.72	.003
Overlap: best friend	.14	.71	.003	.01	.91	>.001	.10	.75	.002
Overlap: others in general	3.89	.06	.08	2.20	.14	.05	.02	.89	>.001

In order to further examine the evidence for mimicry effects in Study 1, we carried out a series of Bayesian ANOVAs using JASP software, version 0.7.5.6 (JASP Team, 2016). Each Bayes factor (BF_{01}) indicates how much more likely the data were to occur under a null model, compared to the model described. The results show that participants' rapport ratings were more likely to occur under a model where mimicry has a main effect on rapport, compared to a null model. This could be considered weak or anecdotal evidence for the effect of mimicry on rapport. There is ambiguous evidence for the main effect of mimicry on self-other overlap towards other people in general. There is weak evidence in favour of the null hypothesis for the main effects of mimicry on trust and similarity ratings. For all other main effects of mimicry and interactions between mimicry and time delay, the Bayes factors indicate substantial to strong evidence in favour of the null hypothesis.

Table S3. Bayes factors for the effects of mimicry and time delay on ratings.

	Model BF_{01}			
Measure	Mimicry main effect	Time main effect	Mimicry main effect + time main effect	Mimicry main effect + time main effect + mimicry x time interaction
Rapport	0.34	2.94	1.01	3.76
Trust	2.01	1.45	3.01	9.01
Similarity	2.34	1.99	4.69	16.85
Smoothness	4.79	2.02	9.85	34.74
Overlap: specific avatar	4.26	0.72	3.21	9.14
Overlap: avatars in general	3.83	1.02	3.79	14.16
Overlap: best friend	4.41	1.44	6.66	22.47
Overlap: others in general	0.89	1.66	1.52	5.21

Note. All models have a prior model probability of 0.2 and include subject.

6. Study 2 exploratory analyses

In order to further examine the evidence for mimicry effects in Study 2, we carried out a series of Bayesian ANOVAs using JASP software, version 0.7.5.6 (JASP Team, 2016). The results (Table S4) show that there is substantial evidence in favour of the null hypothesis for the main effect of mimicry on rapport ratings, and also for trust behaviour in the virtual maze task. There is also weak or anecdotal evidence in favour of the null hypothesis for the main effect of mimicry on liking and trust ratings. There is substantial evidence for null main effects of group on all of the outcomes measured. There is also very strong evidence for null interaction effects between mimicry and group membership on all outcome variables.

Table S4. Bayes factors for the effects of mimicry and group membership on ratings and virtual maze task.

	Model BF_{01}			
Measure	Mimicry main effect	Group main effect	Mimicry main effect + group main effect	Mimicry main effect + group main effect + group x time interaction
Liking rating	2.11	5.79	11.70	49.67
Rapport rating	3.74	3.54	13.26	47.61
Trust rating	1.64	5.61	9.67	30.61
Approach (maze)	5.37	5.45	29.01	34.93
Follow advice (maze)	5.60	5.65	32.01	94.82

Note. All models have a prior model probability of 0.2 and include subject.

In order to check whether participants' experience of co-presence modulated any of the effects in Study 2, we carried out a series of ANCOVAs, controlling for co-presence as a covariate. There were no significant main effects of mimicry on any of our dependent variables when co-presence was included as a covariate in the analysis (Table S5). There were also no significant main effects of group membership or any significant interactions between mimicry and group membership.

Table S5. Effects of mimicry and group on ratings and virtual maze task, controlling for levels of co-presence.

	Main effect of mimicry			Main effect of group			Mimicry x group interaction		
Measure	F(1, 48)	p	η_p^2	F(1, 48)	p	η_p^2	F(1, 48)	p	η_p^2
Liking rating	.05	.83	.002	.14	.71	.004	3.03	.09	.09
Rapport rating	1.14	.29	.03	.12	.73	.004	.35	.55	.01
Trust rating	1.65	.21	.05	.88	.34	.03	.44	.51	.01
Approach (maze)	.17	.68	.005	.12	.73	.004	3.86	.06	.11
Follow advice (maze)	.006	.94	>.001	.008	.93	>.001	.58	.45	.02

It is possible that we did not find a significant effect of mimicry in Study 2 due to fatigue effects: participants might have become bored or disengaged by the time they interacted with the second group of avatars. In order to investigate this possibility, we carried out a series of ANOVAs which only included data from the first group of avatars, i.e. the first half of the experiment. Mimicry was a repeated-measures factor and group membership was a between-subjects factor. There were no significant main effects of mimicry or group membership and no significant interactions between mimicry and group membership in this data (Table S6). This suggests there was not a mimicry effect

from the first two trials which became masked by boredom or fatigue in later trials. Note, however, that this analysis is underpowered for testing between-subjects effects of group membership.

Table S6. Effects of mimicry and group in the first half of Study 2

	Main effect of mimicry			Main effect of group			Mimicry x group interaction		
Measure	*F*(1, 48)	*p*	η_p^2	*F*(1, 48)	*p*	η_p^2	*F*(1, 48)	*p*	η_p^2
Liking rating	.17	.68	.004	0.13	.98	>.001	2.76	.11	.07
Rapport rating	.47	.50	0.01	.016	.90	>.001	.29	.60	.008
Trust rating	3.22	.08	0.08	.013	.91	>.001	.32	.57	.008
Approach (maze)	.01	.93	>.001	2.835	.10	.07	.49	.49	.01
Follow advice (maze)	.03	.87	.001	2.711	.11	.07	.02	.89	.001
